# Comprehensive Analysis on Prognosis and Immune Infiltration of Lysyl Oxidase Family Members in Pancreatic Adenocarcinoma With Experimental Verification

**DOI:** 10.3389/fmolb.2022.778857

**Published:** 2022-04-01

**Authors:** Chao Jiang, Meng Wang, Weikai Yao, Guoyue Lv, Xueyan Liu, Guangyi Wang

**Affiliations:** ^1^ Department of Hepatobiliary Pancreatic Surgery I, The First Hospital of Jilin University, Changchun, China; ^2^ Multi-Organ Transplant Program, University Health Network, Toronto, ON, Canada; ^3^ Department of Pathology, The First Hospital of Jilin University, Changchun, China; ^4^ Cardiovascular Department, China-Japan Union Hospital of Jilin University, Changchun, China

**Keywords:** pancreatic adenocarcinoma, lysyl oxidase, LOX family members, bioinformatics analysis, tumor microenvironment, immune infiltration, prognostic

## Abstract

**Background:** Pancreatic adenocarcinoma (PDAC) is the most aggressive among all solid malignancies with delayed disease detection and limited effective treatment. However, due to the intricate heterogeneity and exclusive tumor microenvironment (TME), the development of effective therapy has been facing enormous challenges. The lysyl oxidases (LOXs) underpin the shaping of the TME to promote cancer growth, metastasis and modulate response to treatment.

**Materials and Methods:** The mRNA expression, prognostic, and clinicopathological data for LOXs in PDAC from multiple open-access databases were summarized and analyzed. The protein expression was verified by immunohistochemistry (IHC). Co-expressed genes of LOXs were predicted and elaborated by LinkedOmics. Functional enrichment analysis of LOXs co-expressed genes was performed using Gene Ontology (GO) and Kyoto Encyclopedia of Genes and Genomes (KEGG). TIMER and TISIDB were applied to analyze the relationship between LOXs expression and immune infiltration.

**Results:** The mRNA expression levels of LOX, LOXL1 and LOXL2 were significantly higher in PDAC, the expression levels of LOXL3 and LOXL4 were contrary in different databases. High mRNA levels of LOX and LOXL2 were associated with advanced PDAC stage, while elevated LOX and LOXL3 expression correlated with high tumor grade. The IHC staining showed higher expression levels of LOX, LOXL1 and LOXL2, lower expression level of LOXL3 in PDAC tissues, while the protein expression of LOXL4 made no difference. Functional enrichment analysis showed a close relationship with extracellular matrix (ECM) regulation, except that LOXL3 and its ligands were highly associated with immune-related functions. Further analysis suggested that LOX and LOXL3 strongly correlated with tumor-infiltrating lymphocytes (TILs), various immune signatures, and immune checkpoints. Finally, survival analysis revealed high LOX and LOXL2 expression predicted worse overall survival (OS), progression-free interval (PFI), and disease-specific survival (DSS).

**Conclusion:** These findings indicated that the LOX family, especially LOX and LOXL2, might have a prospective value in PDAC oncogenesis, and they may become prognostic biomarkers, revealing a promising field in targeted therapy.

## Introduction

Pancreatic adenocarcinoma (PDAC), a highly fatal disease with a poor prognosis, ranks as the seventh leading cause of cancer mortality globally (Sung et al., 2021), is projected to become the second leading cancer-related death in America and Europe in 2030 (Rahib et al., 2014; Quante et al., 2016). However, over the past decades, albeit considerable advances have been made in the diagnosis, surgery, chemotherapy, radiotherapy and systemic therapy, however, due to non-specific symptoms, lacking reliable markers, low surgical resection rate and therapy resistance, only months of overall survival (OS) achieved (Conroy et al., 2011; Winter et al., 2012), with the 5-years survival remaining a grim 10% (Siegel et al., 2021). Therefore, further investigation of pathological features and the pathogenesis of PDAC is imperative for exceptional therapeutic strategies.

Such poor outcomes of PDAC have fueled ongoing efforts to exploit the tumor microenvironment (TME). The TME is a highly complex ecosystem composed of cancer cells, stromal cells, immune cells, and the extracellular matrix (ECM) interaction. Growing evidence has illustrated the pivotal roles of TME components in immune suppression and tumor progression. It is worth noting that the surrounding ECM has long been implicated in accelerating PDAC progression by directly promoting cellular transformation and metastasis. Furthermore, the ECM and its reorganization are of paramount importance in the evolution of both tumor and immune microenvironment and their interaction (Setargew et al., 2021). These alterations may greatly affect the biological behavior of tumor cells and stromal cells, and ultimately influence the prognosis of PDAC patients.

Lysyl oxidase (LOX) family includes five copper-dependent amino enzymes: LOX and LOX-like (LOXL) 1-4 (Molnar et al., 2003; Barker et al., 2012), of which the canonical function is catalyzing collagen and elastin cross-linking (Harris, 1976; Kagan and Li, 2003; Zhao et al., 2018). Lysyl Oxidases (LOXs) contribute to ECM stiffness and homeostasis, and their dysregulation is involved in several diseases, including tissue fibrosis (Al-U’datt et al., 2019; Li et al., 2020) and cancer (Wang et al., 2016; Tan et al., 2020). In reality, the activity of LOXs integrates a complex network within the TME linking the ECM and the immunological components. Intriguingly, LOXs play different roles in various tumor progression, exhibiting either enhancing (Wilgus et al., 2011; Kasashima et al., 2016)or suppressing (Johnston and Lopez, 2018; Zhao et al., 2020) function in different types of human malignancies.

It has been demonstrated that overexpressed LOXs are critically involved in the PDAC progression. Le Calve et al. (Le Calve et al., 2016) found higher mRNA expression of LOX, LOXL1 and LOXL2 in PDAC. Ma et al. (Ma et al., 2019) found that LOX expression might be an independent prognostic factor of progression-free survival (PFI) in PDAC patients. Higher LOXL2 expression elevated the invasiveness of PDAC cells and correlated with lower survival of PDAC patients (Kim et al., 2019). However, the potential roles of LOXs in the PDAC immune response have not been elucidated. Herein, various large-scale open-accessed databases were applied to explore the expression pattern, cancer-related functional aspects, prognostic value, and relationship with the immune infiltrating of LOXs in PDAC.

## Materials and Methods

### Oncomine

Oncomine gene expression array dataset (https://www.oncomine.org/resource/login.html) (Rhodes et al., 2004; Rhodes et al., 2007) was applied to analyze the transcripts levels of LOXs in different cancers. The data were compared by the *t*-test with a cut-off *p*-value of 0.001 and a fold change of 2.

### GEPIA

Gene Expression Profiling Interactive Analysis (GEPIA, http://gepia2.cancer-pku.cn/#index) (Tang et al., 2017) was used to analyze the relative transcriptional expression of LOXs between PDAC tissues and normal tissues. *p* < 0.01 was considered statistically significant.

### Human Protein Atlas

The Human Protein Atlas (HPA, https://www.proteinatlas.org/) (Uhlen et al., 2015; Uhlen et al., 2017) was introduced to explore the direct contrast of protein expression of LOXL1-4 between normal tissues and PDAC tissues based on immunohistochemistry (IHC). The data of LOX were not available in HPA.

### UALCAN

The UALCAN (http://ualcan.path.uab.edu/index.html) (Chandrashekar et al., 2017) was used to explore the protein expression of LOXs in PADC. The protein assembly data relative abundance was obtained from the Pancreatic Ductal Adenocarcinoma (PDAC) Discovery Study of Clinical Proteomics Tumor Analysis Consortium (CPTAC, https://proteomics.cancer.gov/programs/cptac). *p* < 0.01 was considered statistically significant.

### Immunohistochemistry

As the feedback regulation loop of LOXs, the regulation of transcripts level and protein level are not necessarily the same. To further explore the LOXs protein level in PDAC patients, LOXs expression was evaluated through IHC in PDAC tissue and paired adjacent tissue(*n* = 6). The study was approved by the First Hospital of Jilin University Ethics Committee (2019-180). All patients provided written informed consent for the subsequent use of their resected tissues in this study. All clinical investigations were conducted following the principles of the Declaration of Helsinki. The PDAC tissues and paired adjacent tissues were prepared into 4 μm paraffin sections and incubated overnight at 4 °C with rabbit polyclonal antibodies against LOXs (1:150, Novus Biologicals, USA. LOX: NB100-2527; LOXL1: H00004016-D01P; LOXL2: NBP1-32954; LOXL3: NBP2-75964; LOXL4: NBP2-32692). After washing, the sections were conjugated with ImmPRESS anti-Rabbit kit for 30 min (LSBio, USA. LS-J1066). The negative controls demonstrated the specific signals of the primary antibody. Subsequently, all fields were observed under light microscopy. The average integrated option density (AOD) of LOXs was determined using ImageJ software (National Institutes of Health, USA).

### Metascape

Metascape (https://metascape.org/gp/index.html#/main) (Zhou et al., 2019) was applied to explore the enrichment of the co-expressed genes with LOXs categorized by process and pathway. The most statistically significant 20 terms were shown in the diagram. Terms with *p* < 0.01, enrichment factor>1.5, and minimum count three were considered significant. The protein-protein interaction (PPI) was conducted using the Molecular Complex Detection (MCODE) algorithm.

### LinkedOmics Database

The LinkedOmics database (http://www.linkedomics.org/login.php) (Vasaikar et al., 2018), a helpful third-party online tool containing The Cancer Genome Atlas (TCGA) data(Tomczak et al., 2015), was used to identify the co-expressed genes of LOXs. The correlation was expressed by the Pearson coefficient.

### TIMER

Tumor Immune Estimation Resource (TIMER) database (http://timer.cistrome.org/) (Li et al., 2017; Li et al., 2020) was applied to assess the association of LOXs expression with tumor-infiltrating lymphocytes (TILs) via the Gene module and immune biomarker sets (Siemers et al., 2017) (including immune checkpoint gene sets) via Correlation module. The correlation was adjusted by tumor purity and expressed by the Spearman coefficient (Chen et al., 2020).

### TISIDB

TISIDB (http://cis.hku.hk/TISIDB/) (Ru et al., 2019) was introduced to validate the correlation of LOXs expression with the abundance of TILs, and the relationship of LOXs expression with clinical characteristics in PDAC patients. The correlation was expressed by the Spearman coefficient.

### Survival Analysis

The mRNA expression of LOXs in PDAC was obtained from c-BioPortal (www.cbioportal.org) (Cerami et al., 2012; Gao et al., 2013), and the clinical data of PDAC was extracted from integrated clinical data resources(Liu et al., 2018) to perform survival analysis. Patients were divided into two groups based on the median value of mRNA expression.

### Data Visualization and Analysis

The Venn diagram was drawn via a friendly online tool (http://bioinformatics.psb.ugent.be/webtools/Venn/) to show the overlapping co-expressed genes of LOXs; the heatmap was conducted by TBtools (Chen et al., 2020); the KEGG Mapper (Shannon et al., 2003) was applied to conduct the cancer network using the overlapping genes retrieved from UniProt (https://www.uniprot.org/) (UniProt, 2021). The AOD of LOXs was expressed as the mean ± standard deviation. Data were analyzed with a two-tailed Student’s t-test for a parametric test. *p* < 0.05 was considered to indicate a statistically significant difference.

## Results

### High mRNA Expression of LOXs in PDAC Tissues

**FIGURE 1:**
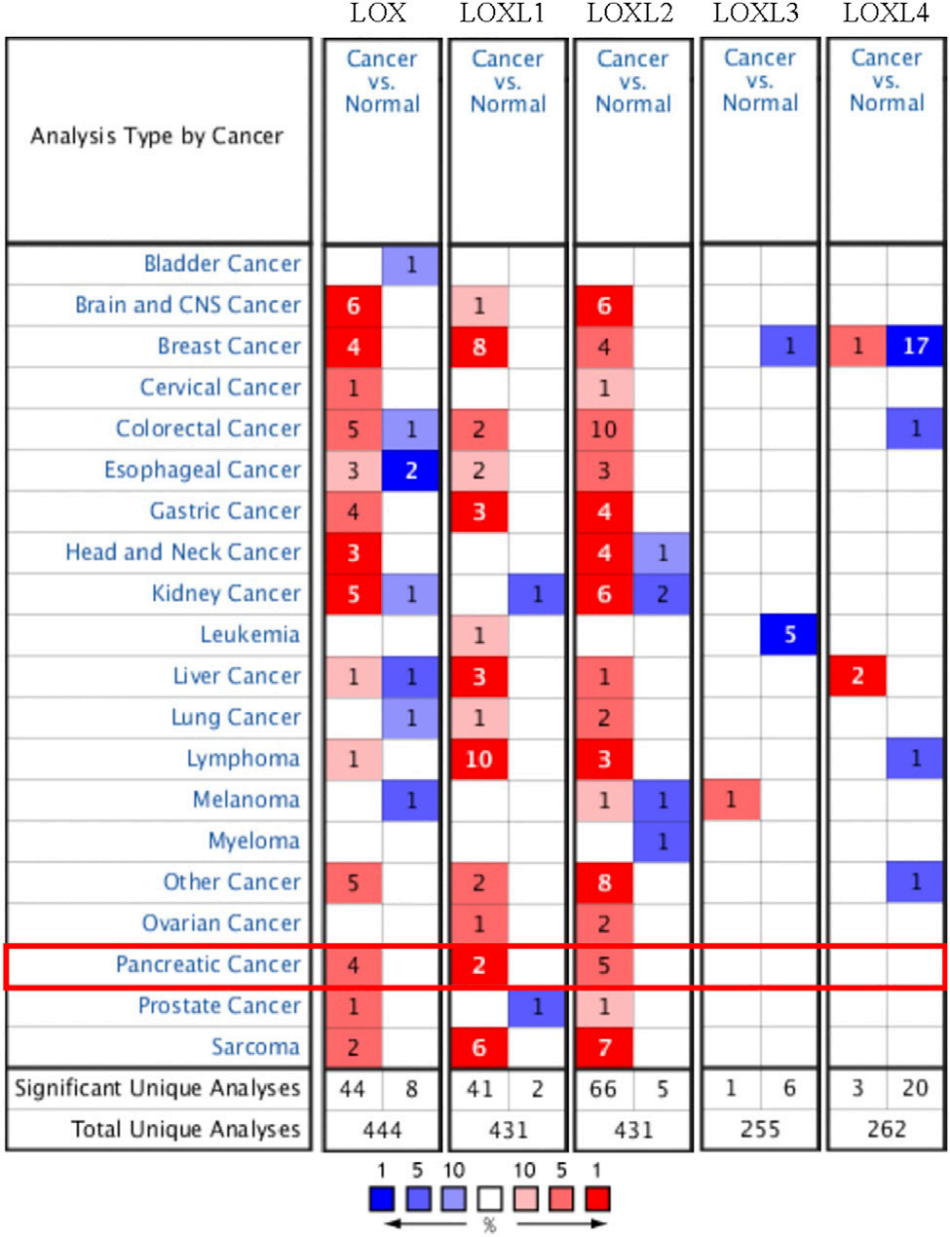
The transcriptional expression of distinct LOX family members in 20 types of cancers (Oncomine database). The numbers in each table represented the studies with statistically significant tumor tissue mRNA overexpression (red) or down-expression (blue), and LOX, LOXL1, and LOXL2 expression were upregulated in PDAC tissues.

The mRNA expression of LOX, LOXL1, and LOXL2 was significantly higher in PDAC tissues than healthy tissues based on the Oncomine analysis (Figure 1 and Table 1). In Logsdon PDAC dataset(Logsdon et al., 2003), LOX and LOXL2 transcripts were upregulated in PDAC tissues by fold changes of 4.433 and 5.235 (*p* = 2.78E-4, 4.56E-6), respectively. Iacobuzio-Donahue (Iacobuzio-Donahue et al., 2003) found a 2.084-fold increase of LOXL1 and a 3.334-fold increase of LOXL2 in PDAC tissues. Moreover, other studies were added to support the upregulation of LOX, LOXL1, and LOXL2 in PDAC (Badea et al., 2008; Pei et al., 2009).

**TABLE 1:** The significant changes of LOXs expression in transcriptional level between PAAD and normal pancreas tissues (Oncomine).

	Type of PAAD *vs.* pancreas	Fold change	*p*-value	*t*-test	PMID
LOX	Pancreatic adenocarcinoma	4.433	2.78E-04	5.864	12750293
	Pancreatic ductal adenocarcinoma	8.835	9.96E-13	9.142	19260470
	Pancreatic carcinoma	2.933	6.00E-4	4.353	15867264
LOXL1	Pancreatic ductal adenocarcinoma	5.245	3.97E-16	11.346	19260470
	Pancreatic adenocarcinoma	2.084	9.01E-04	3.937	12651607
LOXL2	Pancreatic adenocarcinoma	5.235	4.56E-06	7.695	12750293
	Pancreatic carcinoma	4.989	2.63E-07	6.39	19732725
	Pancreatic adenocarcinoma	3.334	5.61E-04	4.255	12651607
	Pancreatic ductal adenocarcinoma	3.172	5.96E-11	7.589	19260470
	Pancreatic carcinoma	3.014	5.13E-4	4.277	15867264

**FIGURE 2:**
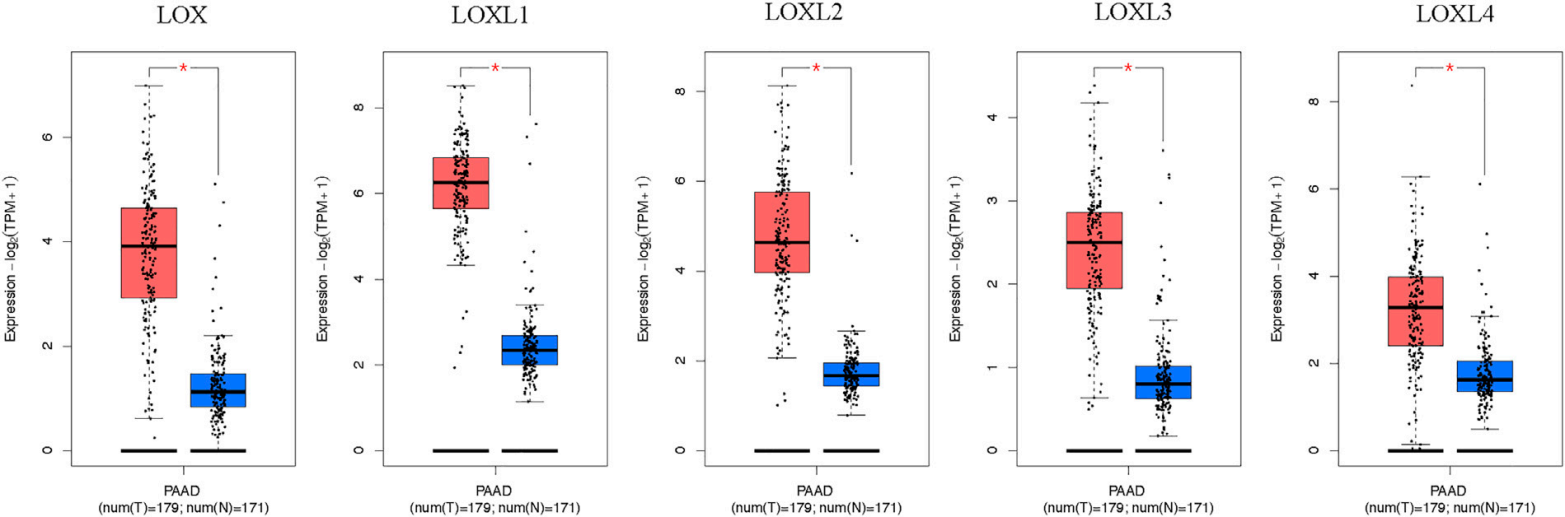
Boxplot showing the expression levels of LOXs in PDAC. The mRNA expression of LOXs was significantly elevated in PDAC than normal pancreas tissues (GEPIA database). The number of normal samples is 171 (blue box), and PDAC samples are 179 (red box), red star means *p* < 0.01.

Furthermore, the mRNA expression patterns of LOXs were further measured by GEPIA based on the TCGA database. The GEPIA analyses showed all LOXs were higher in PDAC than in normal tissues (Figure 2).

### Protein Expression of LOXs in PDAC Tissues

**FIGURE 3:**
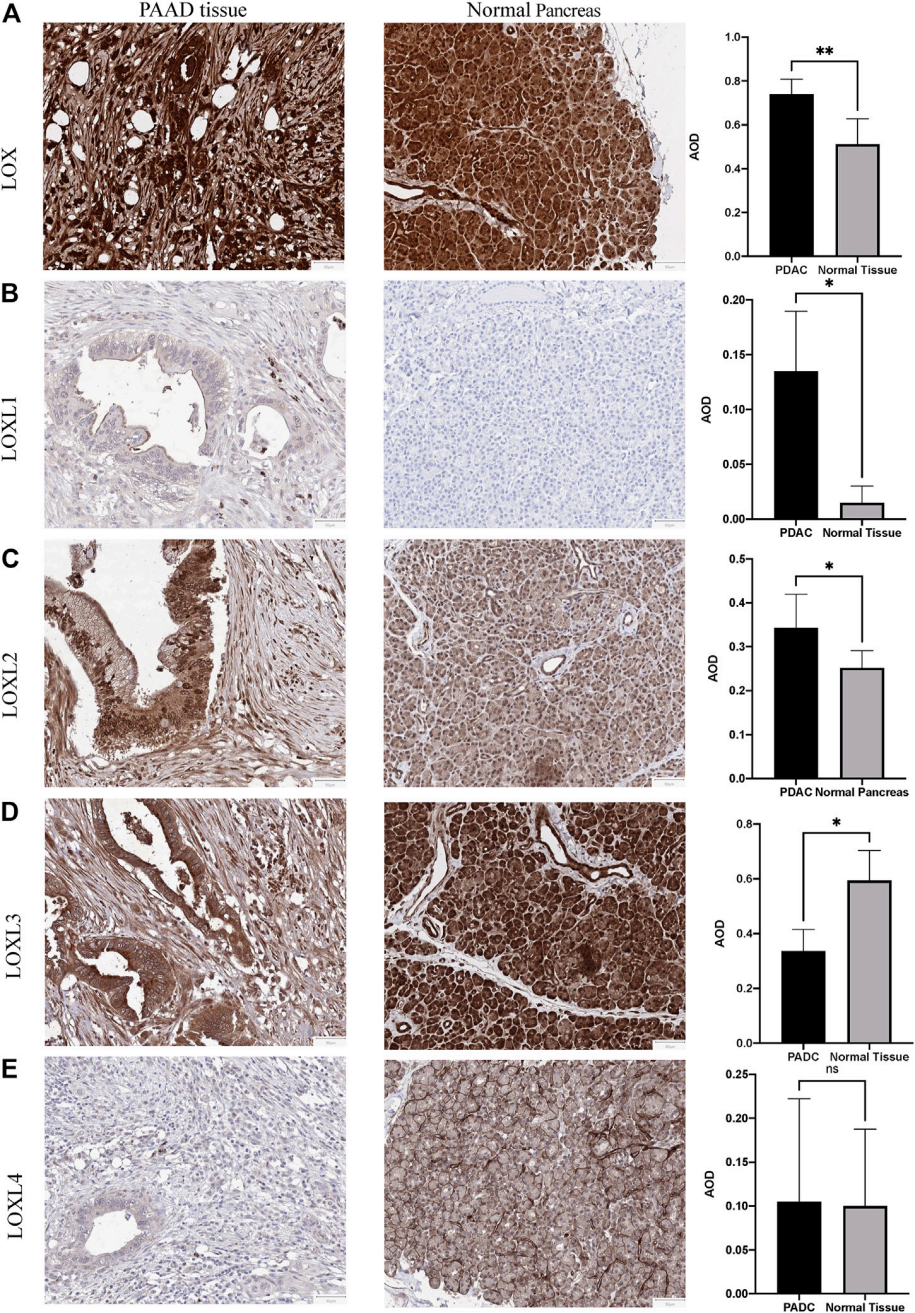
Representative immunohistochemistry of LOXs in PDAC tissues and normal pancreas tissues. **(A)** IHC staining of LOX and quantification showed higher expression in PDAC than normal pancreas tissues. **(B)** IHC staining and quantification showed higher expression of LOXL1 in PDAC. **(C)** IHC staining and quantification showed higher expression of LOXL2 in PDAC. **(D)** IHC staining and quantification showed lower expression of LOXL3 in PDAC. **(E)** LOXL4 expression levels were similar in PDAC and normal tissues. *p < 0.01; NS, no significant difference. *n* = 6, repeated 5 times.

To further explore the protein level in PDAC, IHC was performed in PDAC and adjacent pancreas. The IHC results demonstrated elevated LOX, LOXL1 and LOXL2 protein expression in the PDAC tissues (LOX: *p* < 0.01; LOXL1: *p* < 0.05; LOXL2: *p* < 0.05 Figures 3A–C). However, LOXL3 showed a remarkable decrease in PDAC tissues (*p* < 0.05, Figure 3D), and LOXL4 made no difference (Figure 3E).

**FIGURE 4:**
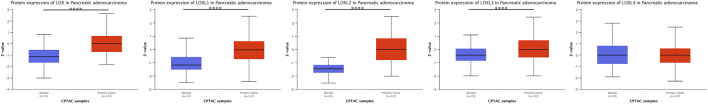
Protein expression levels of LOXs in PDAC tissues and normal tissues (CPTAC database). Protein expression levels of LOX, LOXL1, 2, 3 were significantly up-regulated. ****p < 0.0001.

We also analyzed the protein expression patterns of LOXs in PDAC using HPA and CPTAC datasets. Consistent with our IHC results, HPA results indicated increased LOXL1 and decreased LOXL3 expression in PDAC tissues (Supplementary Figure S1 and Supplementary Table S1), while no significant difference was identified in LOXL2 and LOXL4 compared with exocrine glandular cells in normal tissue. However, the expression of LOLX3 was contrary in different databases. The protein expression level of LOXL3 was identified higher in PDAC tissues than normal tissues verified by CPTAC data (Figure 4).

### Relationship Between LOXs Expression and the Clinical Characteristics of PDAC Patients

**FIGURE 5:**
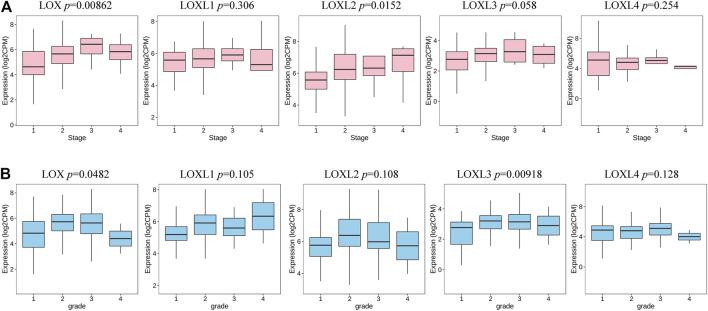
Transcription levels of LOXs in different stages and grades of patients with PDAC (TISIDB). **(A)** The expression of LOX and LOXL2 was highly correlated to the stage of the PDAC. **(B)** The expression of LOX and LOXL3 was significantly associated with tumor grade in PDAC.

We further investigated the association between the mRNA expression of LOXs and the clinical features of PDAC. As indicated in Figure 5, the higher PDAC stage was related to the higher mRNA levels of LOX and LOXL2. Meanwhile, tumor grade was significantly correlated with the expression of LOX and LOXL3.

### LOXs Correlated Genes and Functional Enrichment Analysis in PDAC

**FIGURE 6:**
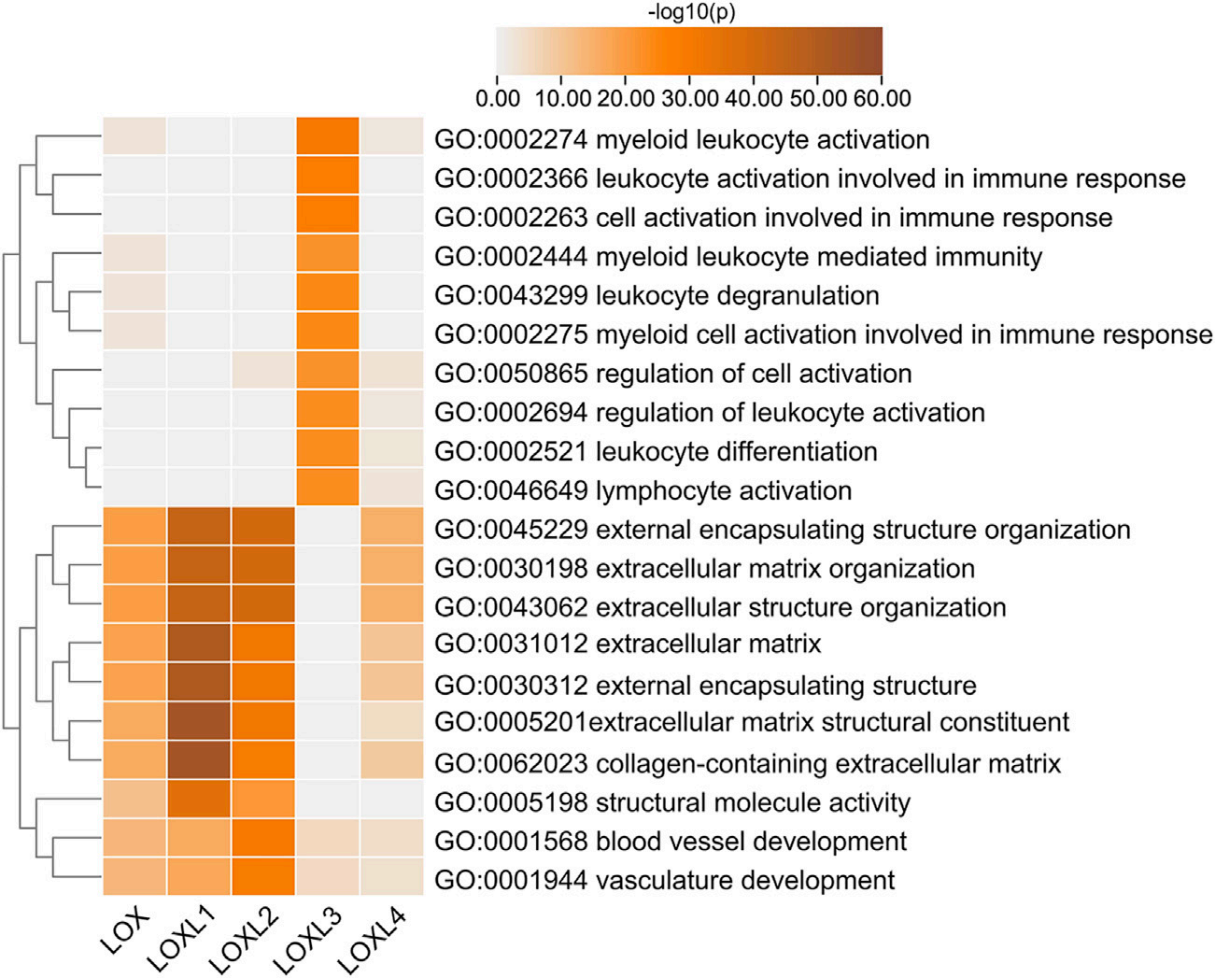
Top 20 statistically significant pathways associated with each LOXs identified by Metascape. Heatmap of the molecular function, biological processes, or pathways enriched with distinct LOX co-expressed genes. The bar color shade was decided by the *p-value*, the deeper the shade the less the *p*-value.

To gain insight into the potential role of LOXs in the PDAC process, we conducted interactome, gene ontology (GO), and Kyoto Encyclopedia of Genes and Genomes (KEGG) enrichment analysis of the top 200 correlated genes of each LOX family member extracted from LinkedOmics. The results revealed that LOX, LOXL1, 2, 4 were mainly involved in ECM regulation, such as extracellular matrix structural constituent (GO: 0005201), collagen-containing extracellular matrix (GO: 0062023), extracellular matrix (GO: 0031012), external encapsulating structure (GO: 0030312), extracellular matrix organization (GO: 0030198), and extracellular structure organization (GO:0043062), while LOXL3 showed a close relationship with immune-related functions, such as myeloid leukocyte activation (GO: 0002274), leukocyte activation involved in immune response (GO: 0002366), myeloid leukocyte mediated immunity (GO: 0002444), myeloid cell activation involved in immune response (GO: 002275), regulation of leukocyte activation (GO: 0002694), leukocyte differentiation (GO: 0002521), and lymphocyte activation (GO: 0046649) (Figure 6 and Supplementary Table S2).

**FIGURE 7:**
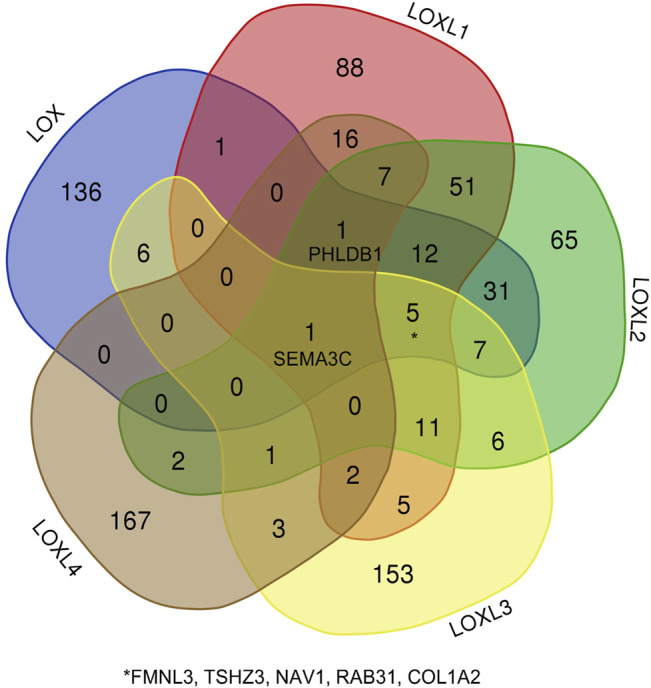
The Venn diagram represents the correlated genes of LOXs based on the Pearson correlation coefficient.

**FIGURE 8:**
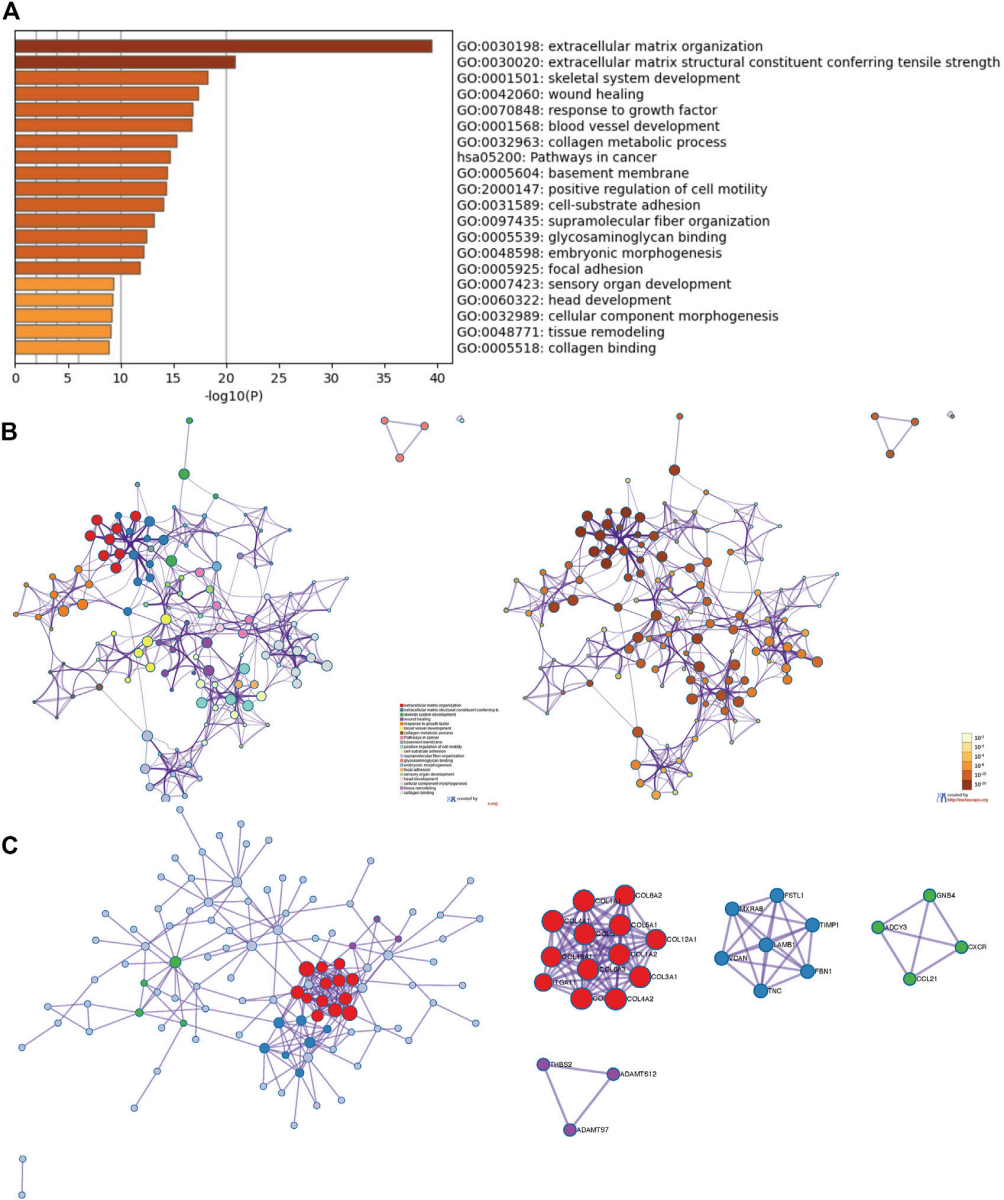
The enrichment analysis and visualized protein-protein interaction (PPI) enrichment analysis of co-expressed genes of LOXs (Metascape). **(A)** Heatmap of enriched terms regarding Gene Ontology analysis colored by *p*-values. **(B)** An interactive network of the top 20 enrichment terms. **(C)** PPI network and densely connected network components are identified by the Molecular Complex Detection (MCODE).

Besides the specific role of each LOX member plays in cancer, the involvement of several members of LOXs has also been illustrated. Thus, the 168 overlapping genes of each LOX member’s top 200 correlated genes were used to investigate the combined function of the LOX family in PDAC (Figure 7). As indicated in Figure 8A, we observed that functions of those genes were enriched in ECM remodeling, such as extracellular matrix organization (GO: 0030198), extracellular matrix structural constituent conferring tensile strength (GO: 0030020), blood vessel development (GO: 0001568), and collagen metabolic process (GO: 0032963). The networks of enrichment terms of LOXs according to cluster-ID were displayed in Figure 8B. Furthermore, the PPI network for LOXs and overlapping co-expressed genes was conducted, wherein the significant densely connected network constituents were assigned in a unique color (Figure 8C).

**FIGURE 9:**
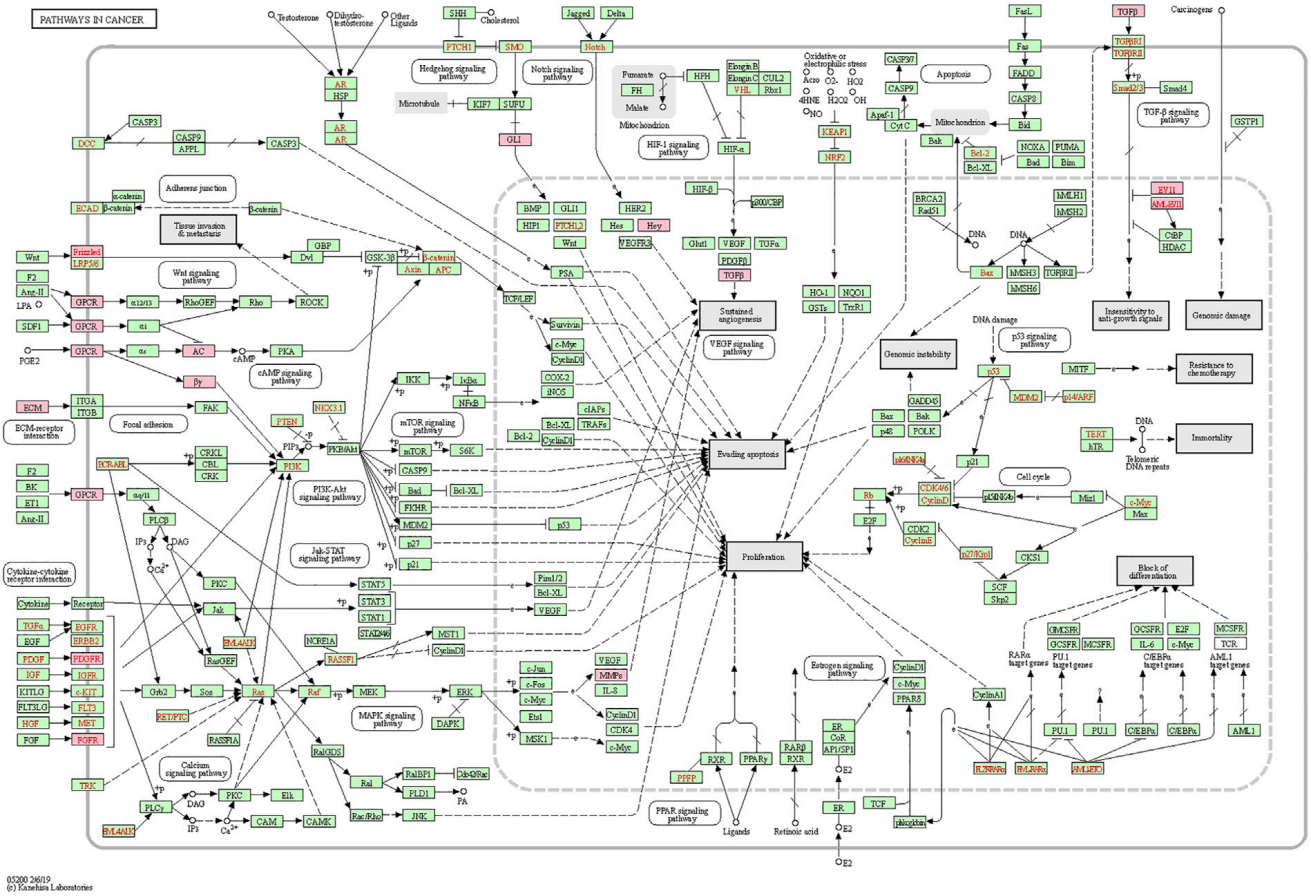
Positional relationship of LOXs co-expressed genes in the cancer network. The overlapping genes were mainly enriched in cancer-related pathways, such as pathways in cancer (hsa05200), PI3K-AKT signaling pathway (has04151), focal adhesion (has04510), ECM-receptor interaction (hsa04512). The relevant positions of the corresponding genes in the pathway are marked with pink circles.

Similar functional enrichment results were achieved using DAVID (https://david.ncifcrf.gov/) (Huang da et al., 2009a; Huang da et al., 2009b) (Supplementary Figure S2). Considering the strong correlation between LOXs-related genes and pathways in cancer (hsa05200) as shown in the Metascape analysis, the KEGG pathway map was generated (Figure 9). The cancer network revealed that those correlated genes were essentially abundant in pathways in cancer (hsa05200), PI3K-AKT signaling pathway (has04151), focal adhesion (has04510), and ECM-receptor interaction (hsa04512), the details illustrated in Supplementary Figure S3.

### Association of LOXs With Immune Infiltration in PDAC

**FIGURE 10:**
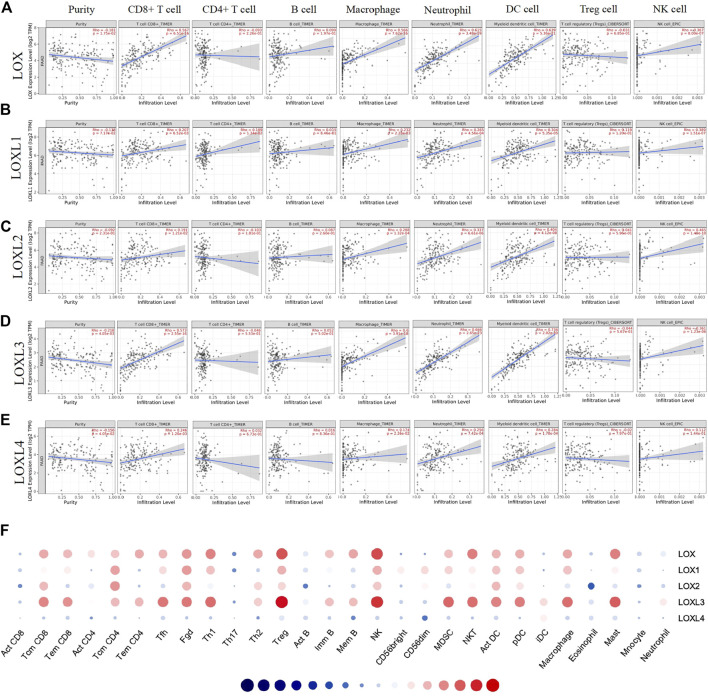
Correlation analysis of LOXs expression and immune infiltration in TME of PDAC. **(A–E)** (TIMER). The correlation between the abundance of immune cells and the expression of **(A)** LOX, **(B)** LOXL1, **(C)** LOXL2, **(D)** LOXL3, **(E)** LOXL4 in PDAC. **(F)** Correlation between the relative abundances of 28 tumor-infiltrating lymphocytes (TILs) and LOXs expression in PDAC (TISIDB).

Considering the critical role of immune cells in the TME contributing to the progression of PDAC and a close relationship of LOXL3 with immune response demonstrated in the current study, we further investigated the correlation of LOXs transcriptional level with immune infiltration in PDAC using the TIMER and TISIDB database. The TIMER results showed that the LOXs expression was negatively related to tumor purity. The mRNA expression of LOX, LOXL1, 3, 4 was remarkably correlated with CD8^+^ T cells. The mRNA expression levels of LOX, LOXL1-3 had a significant correlation with infiltrating levels of macrophages and NK cells. In addition, the LOX family was highly associated with neutrophil and dendritic cell infiltration (Figures 10A–E). It was worth noting that LOX and LOXL3 remarkedly correlated with those TILs.

Then, we explored the correlation between the abundances of TILs and LOXs expression via TISIDB. In line with the TIMER results, the TISIDB analysis demonstrated that high LOX and LOXL3 expression was strongly correlated with the Tcm CD8^+^ T cell, Tem CD8^+^ T cell, Tfh cell, Th1 cell, Treg cell, NK cell, MDSC cell, act DC cell, pDC cell, macrophage, and mast cell infiltration. Meanwhile, the expression of LOXL1, 2, 4 showed a relatively weak correlation with them (Figure 10F and Supplementary Table S3). These results suggested that LOXs, particularly LOX and LOXL3, might affect PDAC by influencing immune infiltration.

### Correlation Between mRNA Expression of LOXs and Immune Biomarkers in PDAC

**FIGURE 11:**
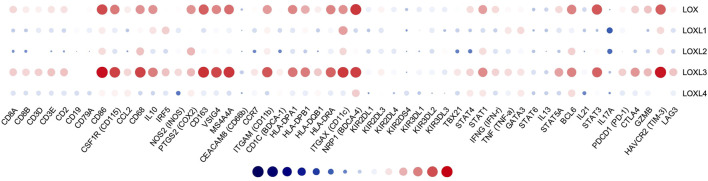
Correlation analysis of LOXs and immune signatures in PDAC (TIMER). The correlation was adjusted by purity.

To better understand LOXs’ crosstalk with immune-related genes, we analyzed the association between the mRNA expression of LOXs and various immune biomarkers using TIMER. The results indicated that the expression of LOX and LOXL3 was highly related to biomarkers of CD8^+^ T cell, T cell, B cell, monocyte, TAM, M1 macrophage, M2 macrophage, neutrophil, DC cell, Th1, Th2, and Th17 in PDAC (Figure 11).

We further explored the immune checkpoint expression level of PDAC cells. The results showed that LOX and LOXL3 were strongly correlated to PD-1, PD-L1, CLTA4, GZMB, TIM-3, and LAG3, while other members of LOX showed no relationship (Table 2). In brief, these results affirmed the role of LOX and LOXL3, especially LOXL3 in the immune response in PDAC.

**TABLE 2:** Correlation analysis of LOXs and immune biomarkers in PAAD (TIMER).

Description	Gene marker	LOX		LOXL1		LOXL2		LOXL3		LOXL4	
		rho	p	rho	p	rho	p	rho	p	rho	p
CD8+T cell	CD8A	0.325	**	0.100	0.300	−0.062	0.533	0.346	***	0.162	0.074
	CD8B	0.281	*	0.095	0.326	−0.067	0.495	0.327	***	0.148	0.107
T cell	CD3D	0.271	*	0.181	0.044	−0.005	0.972	0.340	***	0.153	0.094
	CD3E	0.299	**	0.156	0.085	−0.032	0.781	0.364	***	0.160	0.079
	CD2	0.303	**	0.129	0.167	−0.037	0.732	0.365	***	0.168	0.064
B cell	CD19	0.234	0.005	0.224	0.010	−0.012	0.917	0.236	*	0.152	0.098
	CD79A	0.250	*	0.193	0.030	−0.014	0.905	0.235	*	0.137	0.135
Monocyte	CD86	0.603	***	0.283	*	0.314	**	0.687	***	0.263	*
	CSF1R (CD115)	0.512	***	0.187	0.036	0.160	0.067	0.621	***	0.267	*
TAM	CCL2	0.279	*	0.086	0.379	−0.031	0.789	0.326	***	0.276	*
	CD68	0.461	***	0.255	*	0.368	***	0.619	***	0.129	0.161
	IL10	0.463	***	0.298	**	0.167	0.055	0.505	***	0.124	0.178
M1 Macrophage	IRF5	0.233	0.005	0.340	***	0.140	0.113	0.367	***	0.171	0.059
	NOS2 (INOS)	0.248	*	0.179	0.047	0.225	0.008	0.319	***	−0.130	0.159
	PTGS2 (COX2)	0.460	***	0.244	*	0.388	***	0.384	***	0.275	*
M2 Macrophage	CD163	0.565	***	0.157	0.085	0.212	0.013	0.648	***	0.189	0.033
	VSIG4	0.486	***	0.221	0.011	0.252	0.003	0.611	***	0.182	0.042
	MS4A4A	0.537	***	0.201	0.023	0.208	0.015	0.640	***	0.150	0.103
Neutrophils	CEACAM8 (CD66b)	0.048	0.625	0.016	0.894	0.021	0.855	0.106	0.217	−0.029	0.790
	CCR7	0.249	*	0.143	0.120	−0.079	0.408	0.294	**	0.216	0.014
	ITGAM (CD11b)	0.444	***	0.268	*	0.278	*	0.583	***	0.127	0.239
Dendritic cell	CD1C (BDCA-1)	0.245	0.006	0.135	0.180	−0.085	0.463	0.275	*	0.251	0.006
	HLA-DPA1	0.486	***	0.171	0.075	0.163	0.099	0.522	***	0.192	0.049
	HLA-DPB1	0.411	***	0.256	*	0.099	0.375	0.509	***	0.189	0.052
	HLA-DQB1	0.278	*	0.106	0.310	−0.026	0.842	0.352	***	0.258	*
	HLA-DRA	0.490	***	0.215	0.019	0.195	0.040	0.559	***	0.181	0.067
	ITGAX (CD11c)	0.466	***	0.435	***	0.323	**	0.635	***	0.185	0.059
	NRP1 (BDCA-4)	0.639	***	0.131	0.100	0.251	*	0.625	***	0.345	***
Natural killer cell	KIR2DL1	0.196	0.036	0.085	0.424	0.136	0.189	0.215	0.011	−0.032	0.805
	KIR2DL3	0.265	*	0.169	0.080	0.190	0.047	0.272	*	0.231	0.013
	KIR2DL4	0.192	0.041	−0.001	0.993	0.175	0.071	0.217	0.010	0.040	0.753
	KIR2DS4	0.069	0.588	−0.040	0.744	0.024	0.858	0.024	0.822	0.152	0.143
	KIR3DL1	0.184	0.053	−0.006	0.978	0.027	0.837	0.209	0.013	0.089	0.438
	KIR3DL2	0.201	0.031	0.185	0.052	0.149	0.138	0.207	0.015	0.021	0.875
	KIR3DL3	0.189	0.045	0.057	0.628	0.250	0.006	0.176	0.042	0.013	0.928
Th1	TBX21	0.229	0.008	0.117	0.243	−0.104	0.284	0.293	**	0.136	0.172
	STAT4	0.311	**	0.083	0.420	−0.118	0.219	0.332	***	0.287	*
	STAT1	0.438	***	0.146	0.131	0.311	**	0.476	***	0.292	*
	IFNG (IFN-γ)	0.318	**	0.115	0.250	0.161	0.080	0.332	***	0.148	0.134
	TNF (TNF-α)	0.255	*	0.292	*	0.170	0.064	0.314	**	0.201	0.028
Th2	GATA3	0.255	*	0.318	**	0.218	0.014	0.297	**	0.287	*
	STAT6	0.077	0.450	0.043	0.696	0.078	0.432	0.158	0.063	0.096	0.360
	IL13	0.126	0.185	0.082	0.426	0.019	0.873	0.118	0.174	0.108	0.301
	STAT5A	0.319	**	0.171	0.068	0.078	0.432	0.429	***	0.127	0.209
Tfh	BCL6	0.494	***	0.200	0.030	0.336	**	0.494	***	0.313	**
	IL21	0.137	0.146	0.082	0.424	−0.023	0.844	0.220	0.008	−0.092	0.389
Th17	STAT3	0.581	***	0.058	0.595	0.252	*	0.559	***	0.225	0.012
	IL17A	0.022	0.855	−0.210	0.021	−0.163	0.076	0.050	0.601	−0.046	0.689
T cell exhaustion	PDCD1 (PD-1)	0.263	*	0.215	0.018	0.006	0.956	0.339	***	0.177	0.062
	CTLA4	0.339	***	0.241	0.007	0.086	0.385	0.456	***	0.187	0.046
	GZMB	0.365	***	0.062	0.562	0.122	0.202	0.382	***	0.206	0.024
	HAVCR2 (TIM-3)	0.566	***	0.317	**	0.313	**	0.682	***	0.240	0.007
	LAG3	0.262	*	0.156	0.101	0.123	0.197	0.361	***	0.193	0.038
	CD274 (PD-L1)	0.562	***	0.099	0.196	0.284	**	0.551	***	0.291	**

*p < 0.01, **p < 0.001, ***p < 0.0001.

### Prognostic Value of mRNA Expression of LOXs in PDAC Patients

**FIGURE 12:**
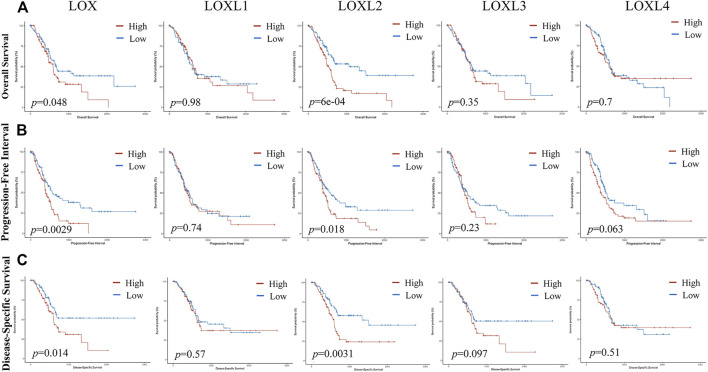
The prognosis value of LOXs expression in PDAC. **(A)** Upregulated LOX and LOXL2 were highly correlated with poor overall survival (OS) in PDAC patients. **(B)** Upregulated LOX, LOXL2, and LOXL4 were significantly associated with inadequate progression-free interval (PFI). **(C)** LOX and LOXL2 expression was negatively related to disease-specific survival (DSS).

As displayed in Figures 12A,B, **C** the mRNA levels of LOX and LOXL2 were significantly correlated with poor OS (*p* = 0.048 and 6e-04), progression-free interval (PFI) (*p* = 0.0029 and 0.018), and disease-specific survival (DSS) (*p* = 0.014 and 0.0031) in PDAC. However, the mRNA levels of LOXL1, 3, 4 showed no relationship with the overcomes in PDAC patients. The mRNA levels of LOX and LOXL2 highly correlated with the outcomes of PDAC patients, indicating LOX and LOXL2 may be served as biomarkers for predicting the prognosis.

## Discussion

As a kind of secreted protein family, LOXs play a pivotal role in PDAC pathogenesis by regulating the TME in two aspects, ECM remodeling and immune-related responses. It has been well known that the TME could significantly affect the behavior of tumor cells and ultimately influence the outcomes of patients (Setargew et al., 2021). The TME of PDAC is characterized by dense desmoplasia and extensive immunosuppression (Neesse et al., 2015; Ren et al., 2018). LOXs are major drivers in the biogenesis of the desmoplastic matrix in cancer. LOXs prevent collagen degradation and improve tissue stiffness through catalyzing the cross-linking process of collagen and elastin, and finally contribute to dense desmoplasia in PDAC. Dense desmoplasia plays a critical role in metastasis and limiting chemotherapy efficacy (Incio et al., 2016; Ren et al., 2018; Schizas et al., 2020). With the increasing interest in anti-stromal therapies, the LOX family has emerged as a potential target. Although the role of the LOX family in tumorigenesis and prognosis of several cancers has been partially confirmed, further bioinformatics analysis of PADC has yet to be performed. In this study, we used multitalented public databases to reveal the dysregulated expression of the LOXs and their relationship with tumor stage and prognosis. As for extensive immunosuppression in PDAC, we found that LOXs, particularly LOX and LOXL3 are closely related to TILs, various immune signatures, and immune checkpoints, however, the function in the immune microenvironment remains unclear which calls for further studies.

Elevated LOX expression has been noted in PDAC tissues compared to adjacent tissues and correlated with poor OS and DSS (Miller et al., 2015; Ma et al., 2019). A high expression of LOX was detected in metastatic pancreatic cancer in the mice model. Conversely, LOX inhibition increased immune cell recruitment, vascularization, and decreased fibrillar collagen (Miller et al., 2015). In line with this finding, Le Calve et al. (2016) found that LOX protein was upregulated in PDAC tissues. Compared to adjacent pancreas tissues, we found higher mRNA expression of LOX in PDAC tissues, which was correlated with tumor stage and grade of PDAC. The IHC showed a higher LOX protein level in PDAC tissues than normal pancreatic tissues. The functional enrichment analysis showed that LOX and its ligands were mainly involved in the ECM regulatory. The transcriptional level of LOX is strongly related to the infiltration of CD8^+^ T cell, macrophage, NK cell, neutrophil, dendritic cell infiltration, as well as various immune biomarkers and immune checkpoints. Furthermore, a high LOX expression was significantly associated with poor OS, PFI, and DSS in PDAC patients. These results together indicate the oncogenic role and prognostic value of LOX in PDAC, as well as the role in the immune response.

LOXL1 transcript was also detected upregulated in PDAC patients and associated with chemotherapy resistance (Le Calve et al., 2016). In our study, the mRNA and protein expression of LOXL1 in PDAC was higher than that in normal tissues. However, the mRNA expression of LOXL1 showed no relationship with tumor stage, grade, and outcomes of PDAC patients. We also revealed the role of LOXL1 in ECM shaping and a close relationship with infiltrating levels of CD8^+^ T cell, macrophage and NK cell, neutrophil, and dendritic cell. However, more studies should be carried out to identify the potential function of LOXL1 in PDAC progression.

Higher LOXL2 expression correlated with clinicopathological features of advanced disease and predicted a worse OS of PDAC patients (Park et al., 2016; Kim et al., 2019). Previous studies have revealed that LOXL2 induced epithelial-mesenchymal transition (EMT) through the PI3K-AKT signaling pathway (Wu and Zhu, 2015) to enhance the invasive and migratory capacity of PDAC cells (Park et al., 2016). In the current study, the expression of LOXL2 was higher in human pancreatic cancer tissues than in normal tissues, which highly correlated to the tumor stage. The functional enrichment analysis showed LOXL2 also had a close relationship with ECM regulation. In addition, LOXL2 expression showed a significant relationship with macrophage and NK cell, neutrophil, and dendritic cell infiltration. And Minici’s study (Minici et al., 2020) has demonstrated LOXL2 is expressed by infiltrating plasmablasts in PDAC. It is worth noting the high expressed LOXL2 predicted an inferior OS, PFI, and DSS in PDAC patients. Consequently, LOXL2 might be a representative biomarker for PDAC. However, phase II clinical trials failed to demonstrate the utility of LOXL2 inhibition in PDAC (Benson et al., 2017), which may be due to cancer-associated fibroblasts (CAFs). The discovery of the tumor-restraining functions of CAFs provides a potential explanation for the unsuccessful clinical trials of therapeutic agents targeting CAFs or stromal components (Van Cutsem et al., 2020). These observations suggest that future therapeutic strategies should avoid generic targeting of tumor-restraining CAF subpopulations in favor of precise reprogramming and normalization of tumor-promoting CAF subsets (Chen et al., 2021).

In this study, overexpressed mRNA of LOXL3 was identified in PDAC tissues and correlated with tumor grade, but showed no relationship with the outcomes of PDAC patients according to the GEPIA database and CPTAC database. Intriguingly, the LOXL3 protein level was found to be downregulated according to our IHC results and HPA database as well, which was controversial. As is known, the expression of LOXL3 was detected mainly in the nucleus correlated with tumor invasion, lymph node metastasis, and poorer prognosis of patients (Kasashima et al., 2018) through interacting with SNAIL and contributing to proliferation and metastasis by inducing epithelial-mesenchymal transition in pancreatic ductal adenocarcinoma cells (Eiseler et al., 2012). Additionally, TGF-induced LOXL3 upregulation in gastric cancer cells suggested that LOXL3 was downstream from the TGF-signaling pathway (Kasashima et al., 2018). However, studies on LOXL3 were mainly based on evidence from cell lines, limited evidence from *in vivo* studies was found. Meanwhile, LOXL3 expression in some tumors was contrary as in some cases LOXL3 was downregulated. Li Ma (Ma et al., 2017) found that LOXL3 was a dual-specificity enzyme involved in STAT3 deacetylation and deacetylimination modulation which reduced the activity of signal transducer and activator of transcription 3 (STAT3). STAT3 activation helps to promote tumor progression (Sun et al., 2021) and is associated with PDAC through PDAC patients’ biopsy study(Laklai et al., 2016). De-activation of STAT3 by LOXL3 would be a protective role in PDAC pathogenesis. Furthermore, unlike other LOX family members, the functional analysis of LOXL3 and its co-expressed genes showed enrichment in the immune response. LOXL3 expression showed a high correlation with CD8^+^ T cell, macrophage, NK cell, neutrophil, and dendritic cell infiltrating levels. In addition, LOXL3 was highly related to biomarkers of CD8^+^ T cell, T cell, B cell, monocyte, TAM, M1macrophage, M2 macrophage, neutrophil, DC cell, Th1, Th2, and Th17. In addition, the LOXL3 expression was positively associated with immune checkpoints. LOXL3 was also found to induce downregulation of Th17 and Treg activation as well (Ma et al., 2017) which play a role in cancer pathogenesis (Marques et al., 2021). Thus, we speculate LOXL3 plays a crucial role in the modulation and recruitment of immune cells and affects the expression of immune signatures in PDAC, and more studies should be carried out to validate.

Accumulating evidence suggest LOXL4 an oncoprotein in several tumors, such as hepatocellular carcinoma (Li et al., 2019), lung cancer (Zhang et al., 2019) and breast cancer (Choi et al., 2017). However, the expression and prognostic role of LOXL4 in PDAC have yet to be investigated. This report demonstrated a higher mRNA expression of LOXL4 in PDAC tissues with no correlation with tumor stage, grade, or outcomes in PDAC patients. The protein expression of LOLX4 showed no difference in PDAC and normal pancreas. The functional analysis of LOXL4 identified similar results to the canonical function of LOXs. In addition, the expression of LOXL4 showed a weak correlation with immune biomarkers. These results together suggest that LOXL4 function in pancreatic tumorigenesis needs to be further determined.

The involvement of one or more LOX members was verified in different cancers. Thus, the LOX family members are likely to present distinct functions specific for each member and partially overlapping activity. We further analyzed the synergy function of LOXs with overlapping co-expressed genes by GO enrichment analysis and KEGG pathway enrichment. GO analysis demonstrated the role of LOXs in ECM regulation. The cancer network showed that changes in LOXs expression mainly affect pathways in cancer (hsa05200), PI3K-AKT signaling pathway (has04151), focal adhesion (has04510), ECM-receptor interaction (hsa04512). These pathways are significantly associated with PDAC progression. Considering the major ECM component in PDAC, the predominant role of LOXs in the ECM remodeling, and involvement in the cancer-related pathways, we speculate that differentially expressed LOXs in PDAC are potential targets for drug therapy.

Current therapeutic strategies aim to deconstruct the surrounding desmoplastic stroma and target immunosuppressive pathways that have largely failed in PDAC. Even immunotherapeutic strategies, such as immune-checkpoint blockade, gained remarkable success in many malignancies that have not yet translated to PDAC (Johnson et al., 2017; Stromnes et al., 2017; Morrison et al., 2018). In addition, ablating the stromal barriers that restrict drug delivery has also demonstrated disappointing and contradictory responses. The key contributor is the multi-faceted functions of the complex TME components (Chronopoulos et al., 2016; Stromnes et al., 2017). Thus, combination strategies that target multiple features of the TME simultaneously might succeed. LOXs inhibitors reduce the fibrotic ECM and desmoplasia and overcome chemoresistance in triple-negative breast cancer by increasing the diffusion of chemotherapeutics into the tumor (Saatci et al., 2020). As demonstrated LOXs played a crucial role in modulating immune cells and affecting the expression of immune signatures in PDAC. Considering the close relationship of LOXs and the immune checkpoints, LOXs inhibitors might also play a role in promoting the anti-tumor effect of immune checkpoint inhibitors. A combination of LOXs inhibitor treatment may improve patient outcomes by priming the TME. However, further preclinical *in vivo* investigations and clinical trials need to be carried out to confirm our speculation.

In conclusion, our research identifies upregulated LOX, LOXL1 and LOXL2 mRNA and protein expression in PDAC tissue. LOX and LOXL2 could serve as novel biomarkers with prognostic significance in PDAC patients. The function of LOXs and their ligands is mainly involved in ECM regulation and pathways in cancer. Intriguing, LOXL3 and its co-expressed genes hold remarked relationship with immune response. Furthermore, a close association between the expression of LOX and LOXL3 with tumor immune infiltration was verified, uncovering the potential molecular mechanism underlying the carcinogenesis in PDAC. Our findings inspire new insight into prognostic biomarkers and new immunotherapeutic targets for PDAC. However, more experimental studies are needed to reveal the link between LOXs and PDAC, thereby promoting the clinical application of LOXs as prognostic indicators and combine therapy targets in PDAC.

## Data Availability

The datasets presented in this study can be found in online repositories. The names of the repository/repositories and accession number(s) can be found in the article/Supplementary Material.
