# Supplementary figures and images for: Comprehensive Analysis on Prognosis and Immune Infiltration of Lysyl Oxidase Family Members in Pancreatic Adenocarcinoma With Experimental Verification
Source: Front Mol Biosci. 2022 Apr 1;9:778857. doi: 10.3389/fmolb.2022.778857 (PMC9010946; doi:10.3389/fmolb.2022.778857)

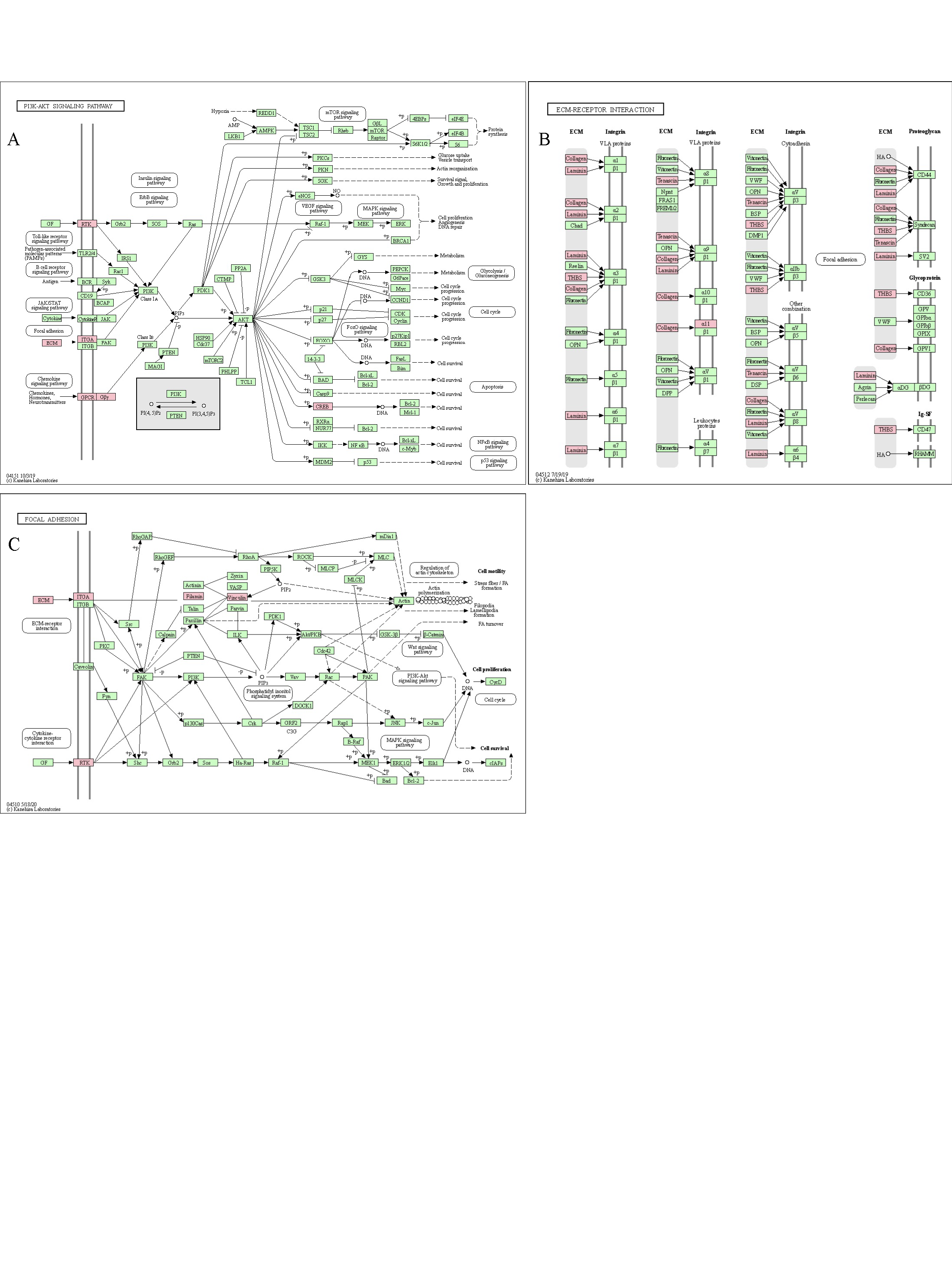

Supplement: Supplementary file 1 [file Image3.TIFF]

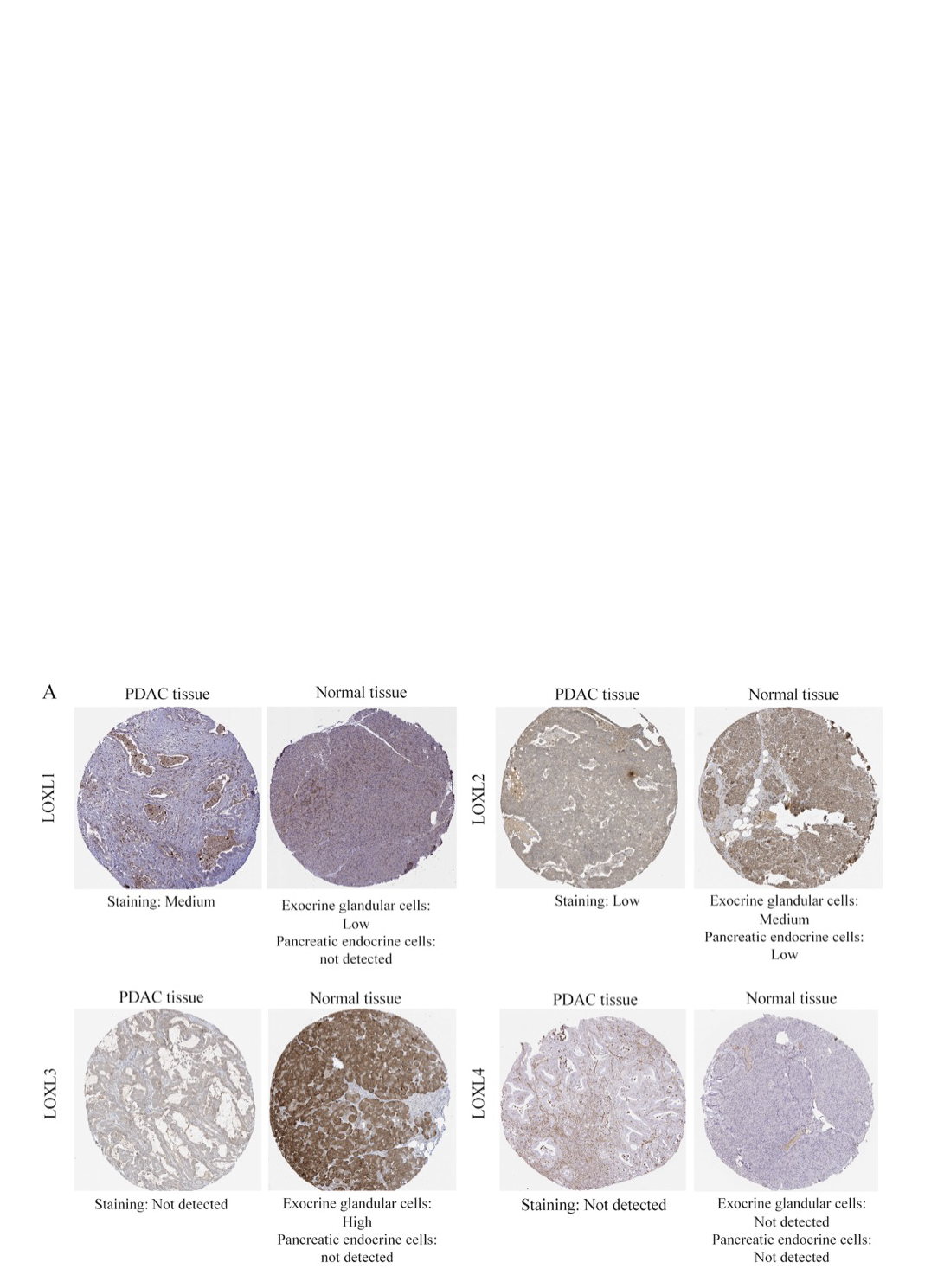

Supplement: Supplementary file 2 [file Image1.TIFF]

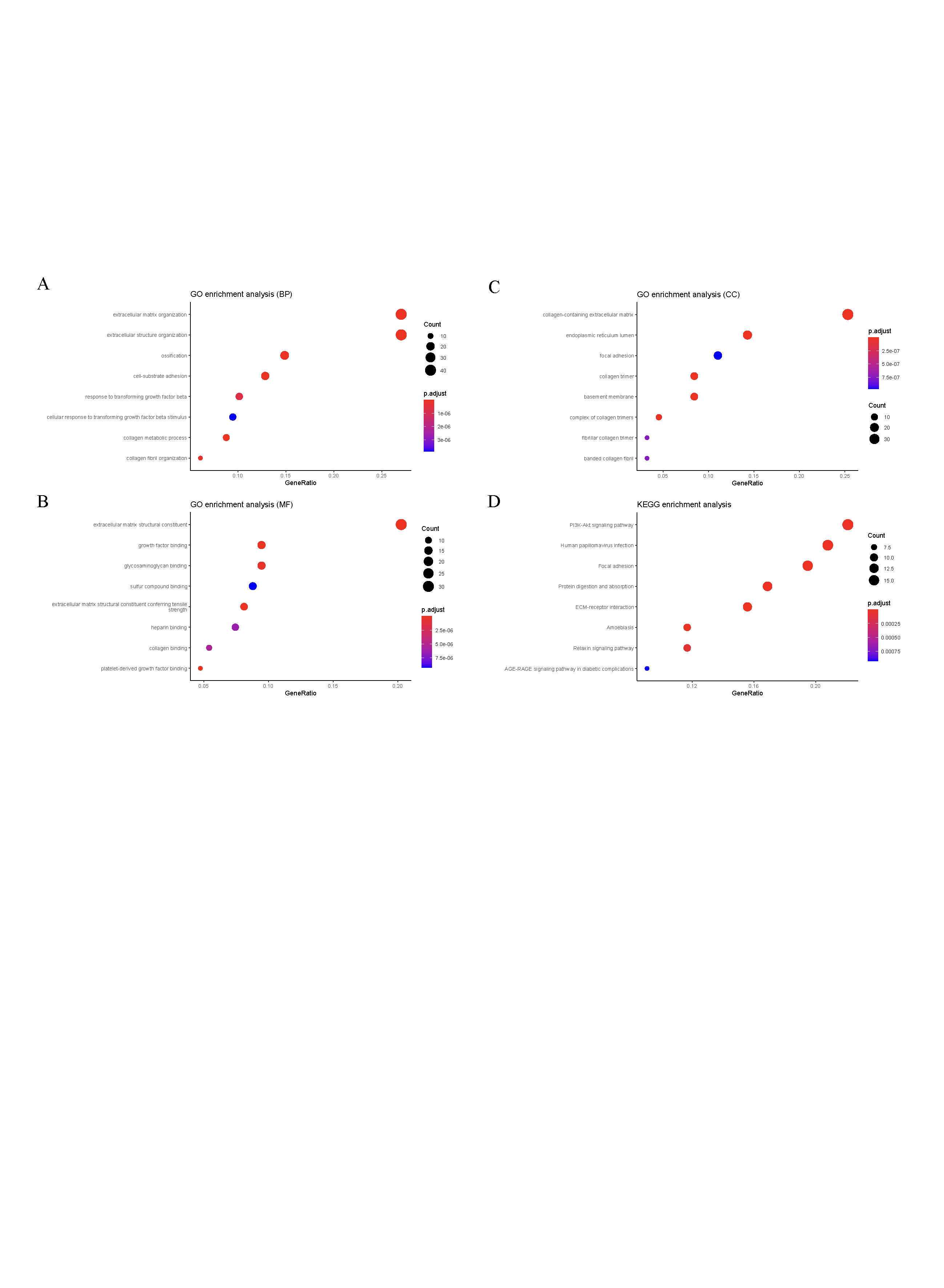

Supplement: Supplementary file 3 [file Image2.TIFF]
